# Mental health services during the war in Ukraine: 2-years follow up study

**DOI:** 10.1186/s13033-025-00667-9

**Published:** 2025-03-28

**Authors:** Irina Pinchuk, Yulia Yachnik, Ryunosuke Goto, Norbert Skokauskas

**Affiliations:** 1 Institute of Psychiatry, Taras Shevchenko National University of Kyiv, Kyiv, Ukraine; 2 Ukrainian Psychiatric Association, Kyiv, Ukraine; 3 Department of Pediatrics, The University of Tokyo Hospital, Tokyo, Japan; 4 Department of Biomedical Data Science, Stanford University, Stanford, CA USA; 5 Regional Centre for Children and Youth Mental Health and Child Welfare - Central Norway, IPH, Norwegian University of Science and Technology, RKBU Midt-Norge, NTNU, Postboks 8905 MTFS, Trondheim, NO-7491 Norway; 6 World Psychiatric Association (WPA), Geneva, Switzerland

**Keywords:** Humanitarian health, Global mental health, Health services research, Ukraine, Psychiatric hospitals, Hospitalizations, Trauma, War

## Abstract

**Background:**

Chronic war exposure causes resource shortages, disrupts care for existing health issues, and heavily impacts mental health, increasing the risk of trauma-related psychiatric disorders. Using longitudinal data collected from psychiatric hospitals across Ukraine between January 2022 and May 2024, we aimed to evaluate the functioning and role of these institutions in delivering mental health care during the ongoing war.

**Methods:**

We conducted a second follow-up nationwide longitudinal study of Ukrainian inpatient mental health facilities during the Russian invasion that began in February 2022. Responses were obtained from the heads of 34 inpatient mental health facilities. This paper focuses on 25 facilities that participated in all three consecutive surveys, representing 41% of all psychiatric hospitals in Ukraine. Information on hospitalizations, as well as data on the number, displacement, and injuries of staff as of April 2024, was gathered and compared with findings from previous surveys.

**Results:**

The number of psychiatric hospitalizations increased two years after the onset of the full-scale war in Ukraine compared to both the pre-war period and six months after the invasion began (433.4 in January 2022, 397.5 in April 2022, and 552.0 in April 2024 per month, respectively). The average proportions of hospitalizations attributed to psychological war trauma across the study waves were 12.2% (January 2022), 13.5% (April 2022), and 17.3% (April 2024), with the differences not reaching statistical significance. The average number of psychiatrists, social workers, and junior nurses per facility declined steadily over the study period. As of April 2024, 21.7% of medical workers in the surveyed facilities had been displaced, and 0.5% had sustained injuries.

**Conclusions:**

The number of psychiatric hospitalizations two years into the full-scale war in Ukraine has risen, exceeding both pre-war levels and those recorded six months after the invasion. While hospitalizations related to war trauma have increased, their proportion has remained relatively stable, indicating a sustained demand for trauma-related care.

## Introduction

Russia’s war on Ukraine continues to cause widespread devastation, including civilian casualties, internal and external displacements, deportations, ongoing trauma, occupation and destruction of infrastructure [1–5]. The World Health Organization (WHO) states that one in four Ukrainians is at risk of developing mental illnesses, such as post-traumatic stress disorder (PTSD), depression, psychoactive substance use, and somatic problems, as a result of the war [6]. It is expected that 1–2 million Ukrainians will develop moderate mental health disorders requiring mental health services, 2–3 million will develop mild mental disorders requiring low-intensity psychological assistance, and 18 million people will be at risk of developing mental disorders, requiring self-help skills, hotline referrals, community support, etc [7].

Acknowledging the significant mental health challenges faced by the population due to the war, Ukrainian decision-makers have prioritized mental health support. For example, in 2022, the First Lady launched the nationwide mental health program “How are you?” [8]. By 2023, the government established the Coordination Centre of Mental Health [9] and adopted key initiatives like the “Operational Roadmap” and the National Action Plan to prioritize mental health during and after the war [10–12]. Ukraine’s mental health system has historically focused on inpatient care, with 89% of the mental health budget allocated to it until 2017 [13]. Despite efforts to decentralize care under the “Concept for the Development of Mental Health Care in Ukraine until 2030,” supported by WHO [13], alternative services remain underdeveloped due to resource and infrastructure gaps and psychiatric hospitals so far continue to be the backbone of mental health services [14].

Our previous study revealed that the Russian invasion severely damaged Ukraine’s mental health services, with acute staff shortages and high hospitalization rates for war trauma, especially in conflict zones and occupied eastern regions [14, 15]. Ukraine has 27 (including Crimea and Sevastopol) regions, each with one main psychiatric hospital. Many regions have several other psychiatric hospitals in addition to the main hospital. The first wave of our study included responses from the heads of 32 facilities (25 leading regional facilities and 7 smaller regional facilities; response rate 86.5%), representing 52.5% of all psychiatric hospitals (62) in Ukraine. Facility directors provided information on mental health services at their institutions for January 2022 (pre-invasion, retrospectively during the first wave), April 2022 (first wave, data collected May 2–June 2, 2022), July–September 2022 (second wave, data collected August 16–September 3, 2022), and May–June 2024 (third wave, data collected May 2–June 2, 2024). In the second wave, 30 of these facilities participated again, accounting for 49.2% of all psychiatric hospitals in the country, providing valuable longitudinal insights. Our study found consistent staff shortages across Ukraine in 2022, despite similar rates of war trauma hospitalizations (11.6% in July vs. 10.2% in April). These hospitalizations became more dispersed as displaced individuals returned to their original locations [15]. We found that Ukraine’s inpatient mental health services, still central to its healthcare system, suffered severe damage in areas targeted by Russian attacks. These challenges compounded an already vulnerable system, which struggled to meet substantial mental health needs even before the war [14, 15].

Chronic war exposure causes resource shortages, disrupts care for existing health issues, and heavily impacts mental health, increasing the risk of trauma-related psychiatric disorders. We considered a follow-up study (third wave) essential to monitor the evolving mental health needs and workforce displacement, with the goal of ensuring sustained access to mental health care. Utilizing longitudinal data collected from psychiatric hospitals across Ukraine between January 2022 and May 2024, we aimed to describe changes in health services provided by psychiatric hospitals in Ukraine during the ongoing war.

## Methods

As with the first two waves, we reached the heads of each of the main psychiatric hospitals in 25 (all regions in Ukraine excluding Crimea and Sevastopol) regions of Ukraine via online messaging, e-mails, and phone calls. In addition to answering about their own hospitals, the directors of the main hospitals were asked to distribute the questionnaires to other psychiatric hospitals in the region. The directors of each facility consented to participate by completing the study questionnaire.

Data was collected from May 28 to July 3, 2024. As with previous two waves, heads of the hospitals were asked about the number of inpatient beds, total hospital admissions, hospital admissions related to war trauma (referring to any mental illnesses secondary to exposure to war), number of staff (psychiatrists, nurses, junior nurses, psychologists, and social workers), number of injured workers, number of displaced workers, whether their facility was directly occupied by Russian forces, the humanitarian aid they received (medical supplies, food, etc.), and additional needs that they may have.

Comparisons between pre-full scale war and the study period, as well as across survey waves, were performed using Wilcoxon signed-rank tests. Percent changes in hospitalizations and the proportion of hospitalizations related to war trauma between waves were calculated by weighting these percentages based on the number of hospitalizations per facility in January 2022. Similarly, the percentages of injured and displaced workers were weighted according to the total number of medical staff per facility as of January 2022. In addition, we analyzed changes in the number of psychiatrists across the three study waves relative to the baseline. Finally, we mapped the primary patient transfer routes during the full-scale war, using arrows to indicate the direction of transfers. For this, healthcare institutions provided a list of facilities to which they transferred patients and from which they received patients during the specified period. The aggregated data were represented in a diagram, where blue indicates the origin of the patient transfer and red represents the destination. This figure does not provide information on the number of patients transferred. Finally, we plotted the numbers of mental health facilities across five regions of Ukraine (North, East, Central, South, and West), color-coded by whether they were damaged or not damaged during the war (Fig. 4). To this end, we asked hospitals to indicate whether there had been any material damage to their facilities since the beginning of the full-scale war and to describe the damage in an open-ended question. According to the responses, the damage ranged from broken windows to the destruction of entire buildings. The diagram only shows the fact of damage without specifying its extent. The study was approved by the ethics committee of Taras Shevchenko National University of Kyiv’s Institute of Psychiatry (No. 6/16.08.2022). This study was conducted in accordance with the ethical principles outlined in the Declaration of Helsinki, ensuring the protection of participants’ rights, safety, and well-being. All analyses were conducted using Python version 3.12.7. The statistical significance level was set at α = 0.05.

## Results

In this follow up study, we obtained responses from heads of 34 hospitals. These hospitals represented 22 regions of Ukraine, and 27 were regional facilities. 25 facilities that participated in all three consecutive surveys, accounting for 41% of all psychiatric hospitals in Ukraine, were included in the analysis (Table 1).

**Table 1:** Basic characteristics of psychiatric hospitals (*n* = 25)

	January 2022 (Baseline)	April 2022	June, 2022 (1st follow up)	April,2024 (2nd follow up)
Number of inpatient beds, mean (SD)	435.8 (277.7)	435.8 (277.7)	408.1 (275.8)	415 (337.0)
Hospitalizations, mean (SD)	433.4 (298.1)	347.4 (306.6)	397.5 (331.2)	552.0 (446.2)
Increase in hospitalizations compared to baseline (%)	-	-19.8%	-4.5%	32.7%
Percent of hospitalizations related to psychological trauma due to war (%)	-	12.2%	13.5%	17.3%
Number of psychiatrists, mean (SD)	40.0 (27.3)	36.1 (24.8)	33.4 (24.4)	30.9 (23.6)
Number of nurses, mean (SD)	161.4 (122.5)	145.5 (122.7)	136.9 (104.8)	142.4 (110.9)
Number of junior nurses, mean (SD)	177.8 (115.2)	159.4 (111.8)	160.3 (113.4)	148.2 (114.2)
Number of psychologists, mean (SD)	14.7 (39.3)	6.2 (5.6)	6.6 (6.4)	6.7 (5.4)
Number of social workers, mean (SD)	2.1 (2.2)	2.6 (4.1)	1.5 (1.7)	1.1 (1.2)
Injured workers out of total medical workers (%)	-	0.5%	3.8%	0.5%
Displaced workers out of total medical workers (%)	-	9.4%	8.8%	21.7%

Legend: Facility-level characteristics were expressed as mean (standard deviation) or as weighted percentages. A negative percentage increase in hospitalizations represents a decrease in hospitalizations compared to baseline

In April 2024, the average number of inpatient beds per facility was 415. The changes in the number of beds between third wave of the survey and the baseline, as well as the second way were not statistically significant (number of inpatient beds per facility third wave vs. second wave 408.1 vs. 415.0, Wilcoxon signed-rank test, *p* = 0.619; number of inpatient beds per facility third wave vs. baseline 435.8 vs. 415.0, Wilcoxon signed-rank test, *p* = 0.569) (Table 1).

**Fig. 1:**
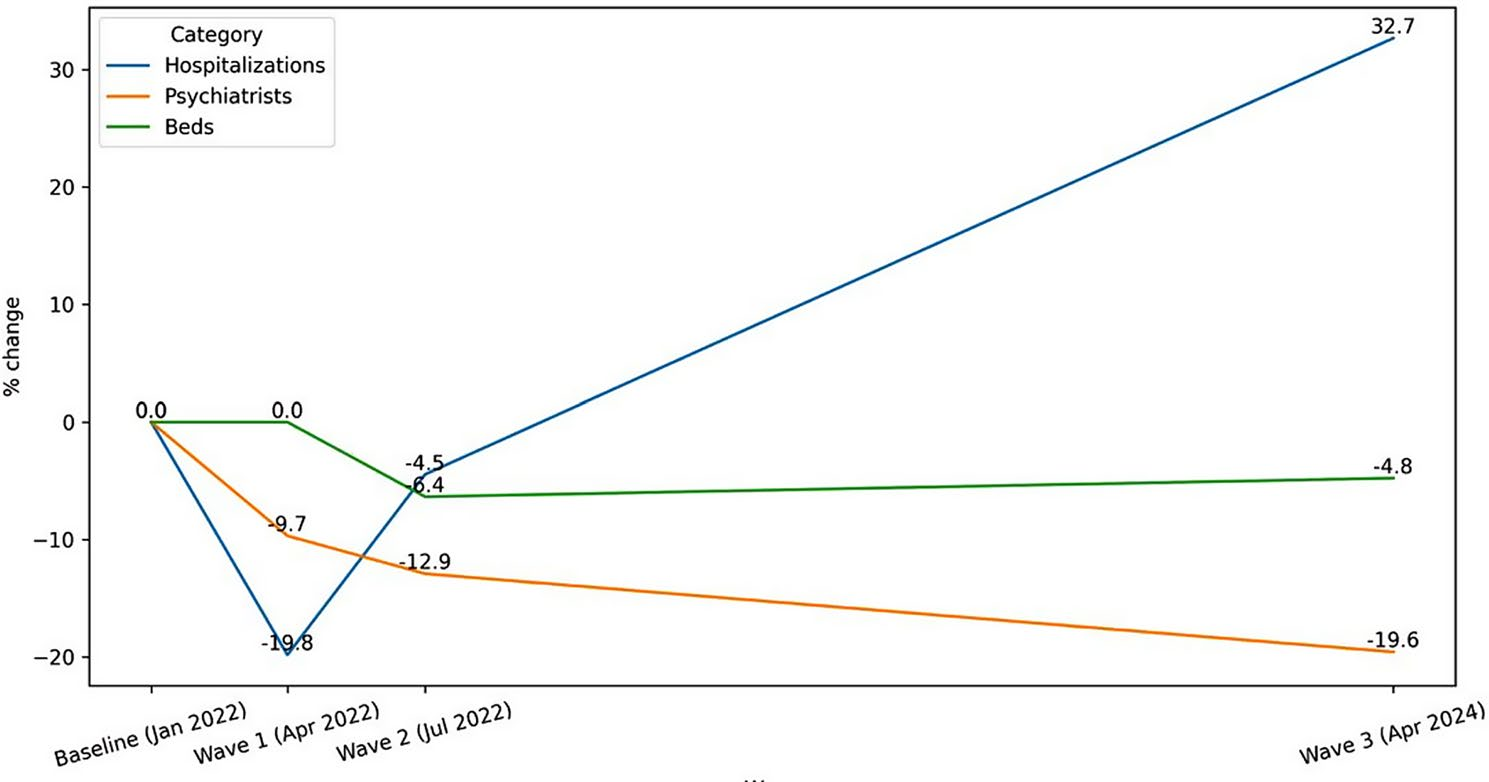
Changes in hospitalizations, number of beds and number of psychiatrists compared to baseline (%)

The number of hospitalizations saw a statistically significant rise compared to the second wave (397.5 vs. 552.0 per month, Wilcoxon signed-rank test, *p* = 0.001), particularly in central, northern, western, and southern regions of Ukraine. Additionally, a statistically significant difference in hospitalizations was observed between 2024 and baseline (433.4 vs. 552.0 per month, Wilcoxon signed-rank test, *p* = 0.035). Hospitalizations related to psychological war trauma due to war remained a substantial proportion of total admissions, with an upward trend noted in 2024. Nonetheless, the increase in percentage of war trauma-related hospitalizations between July 2022 and April 2024 did not reach statistical significance (Wilcoxon signed-rank test, *p* = 0.344) (Fig. 1).

**Fig. 2:**
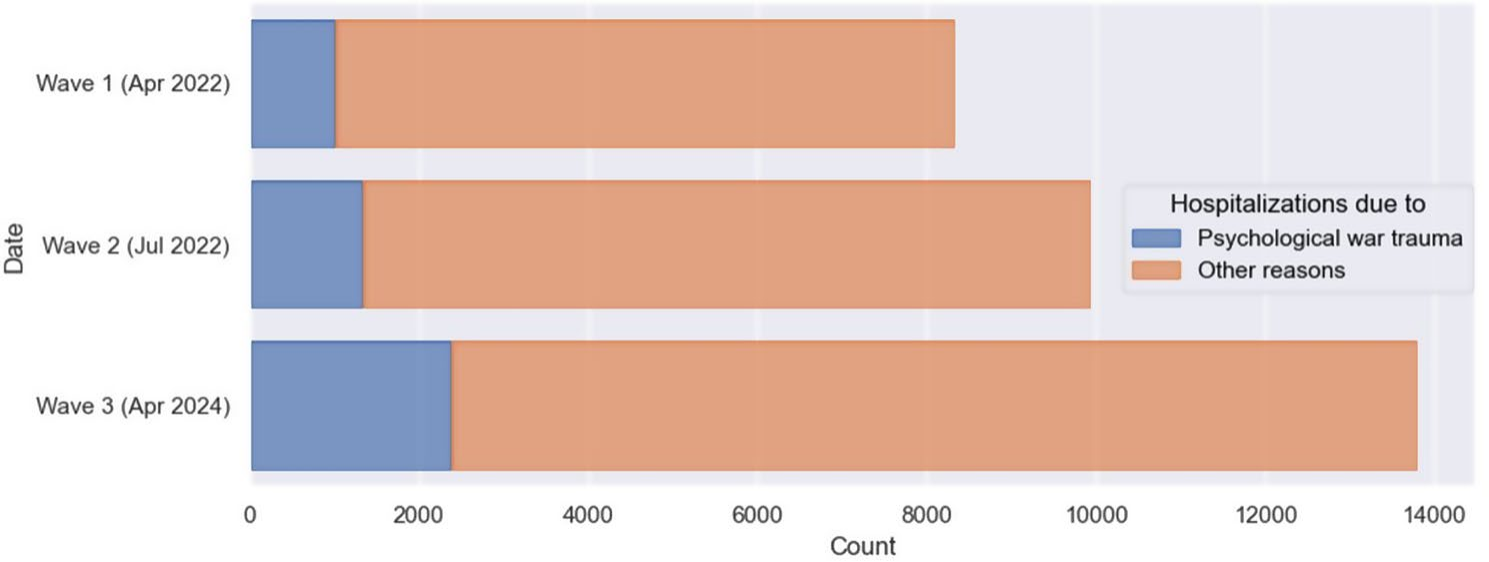
Average proportions of hospitalizations due to psychological war trauma among total number of hospitalizations across different waves

The overall increase in hospitalizations was attributable not only to war trauma but also to other causes (Fig. 2).

The average number of psychiatrists per facility declined continually over the study period, decreasing from 40.0 at baseline to 33.4 in July 2022, and further to 30.9 in April 2024. This decline was statistically significant when compared to baseline (Wilcoxon signed-rank test, *p* = 0.003). However, the reduction from July 2022 to April 2024 was not statistically significant (Wilcoxon signed-rank test, *p* = 0.165).

In addition, the average number of social workers and junior nurses per facility decreased significantly by April 2024 compared to pre-war levels, with reductions from 177.8 to 148.2 for social workers (*p* = 0.009) and from 2.1 to 1.1 for junior nurses (*p* = 0.022). The changes in the average number of psychologists and nurses per facility were not statistically significant compared to before full-scale invasion levels (*p* = 0.10 and 0.12, respectively).

As of April 2024, 21.7% of medical workers in the surveyed facilities had been displaced, and there was an additional 0.5% of sustained injuries. Displacement was particularly common in the eastern, southern, and central regions of Ukraine. Despite these challenges, facilities across the country continued to recruit new staff throughout 2024.

**Fig. 3:**
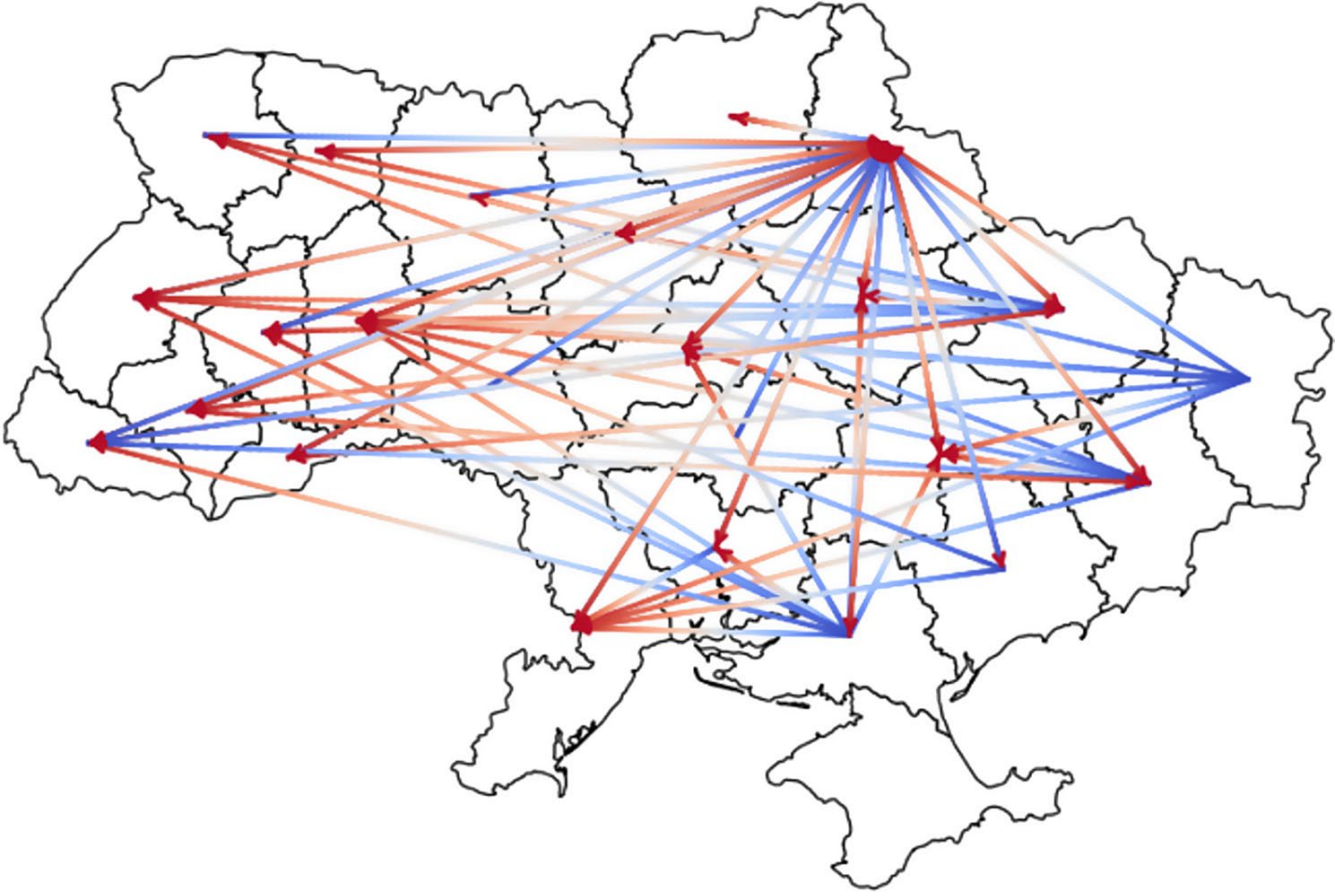
Inter-regional patient transfers among mental health facilities in Ukraine due to military actions (as of 2024)

**Fig. 4:**
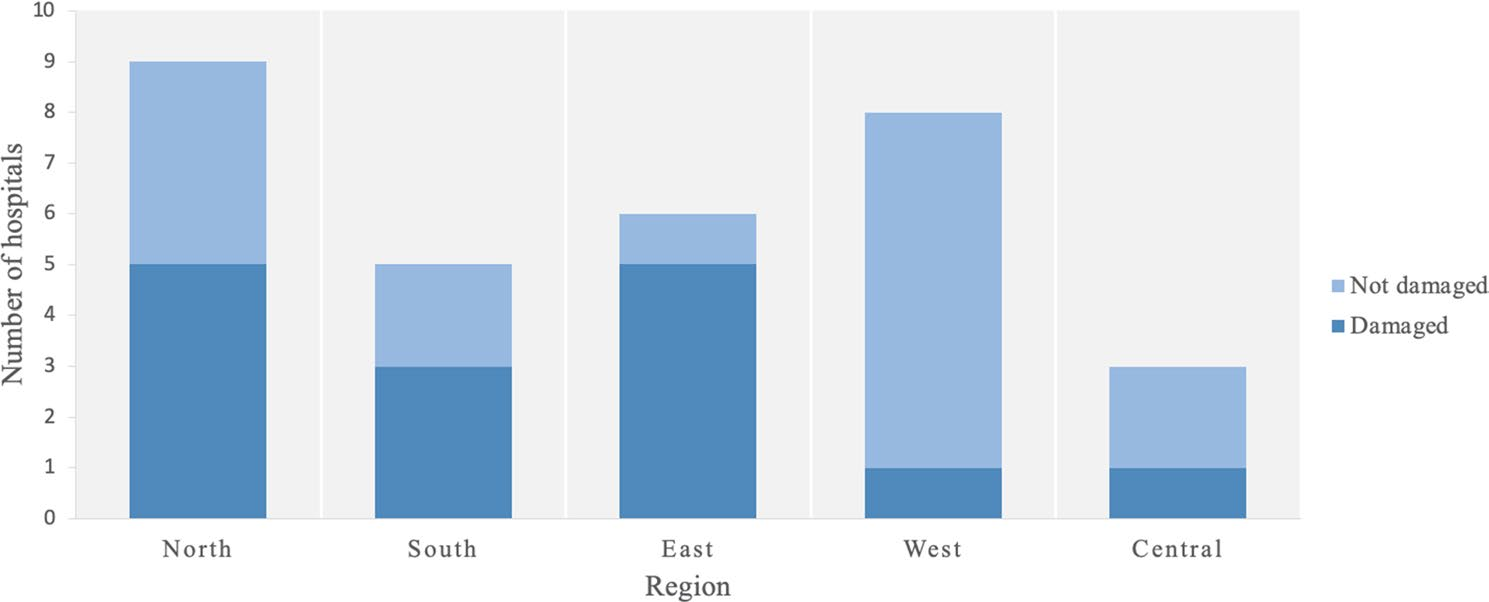
Geographical distribution of damaged psychiatric hospitals in Ukraine due to military actions (as of April 2024)

The war also led to the relocation of many patients to other hospitals. Figure 3 illustrates inter-regional patient transfers among mental health facilities in Ukraine caused by military actions. The majority of patient transfer routes originated from the eastern and southern regions (Fig. 4). The aggregated data were represented in a diagram, where blue indicates the origin of the patient transfer and red represents the destination.

Additionally, nearly half (48.4%) of the hospitals reported damage due to military actions, with the most severe damage concentrated in the eastern and northern regions of Ukraine. Data on the extent of damage were derived from responses to an open-ended question about the degree of facility damage since the onset of the full-scale war. The most severe damage, as described by respondents in phrases such as “entire building completely destroyed,” “several buildings damaged but suitable for use after repairs,” “over 80% of structures destroyed,” or in approximate material loss valuations (e.g., “over 10 million Ukrainian hryvnia”), was recorded in the eastern and northern regions near the combat zone. However, instances of damage were documented across all regions (Fig. 4).

## Discussion

Our study demonstrates a marked increase in psychiatric hospitalizations two years after the onset of the full-scale war in Ukraine, compared to both the pre-war period and six months after the war began. Hospitalizations related to war trauma continue to rise. Additionally, there is a noticeable increase in hospitalizations related to non-war-related psychiatric conditions, which may indicate a broader mental health crisis triggered by the ongoing conflict.

These findings align with prior studies that have documented a surge in war-related mental health problems during wartime [16–19]. Concurrently, there is often an increase in the prevalence of non-war-related psychiatric disorders during war, possibly due to the severe strain on mental health services and the overwhelming stress experienced by affected populations, as well as exacerbation of pre-existing mental health conditions [20–22]. Previous studies have shown that the mental health effects of emergencies and war can persist for years, if not decades [23, 24]. While prolonged stress may, in some cases, foster the development of new coping mechanisms and resilience [25, 26], individuals with pre-existing vulnerabilities may require more intensive and sustained mental health support [27, 28]. This may also explain the total increase in hospitalizations over time and, particularly, the increase in hospitalizations unrelated to psychological trauma that was observed in our study.

Along with that, the ongoing war has severely impacted Ukraine’s psychiatric service infrastructure, which continues to suffer from direct attacks, not only in frontline regions but also across the country. And although we observe a trend of transferring patients from the eastern, northern, and southern regions to the relatively safer central and western regions, none of the regions in Ukraine can be considered completely safe. Furthermore, it was impossible to obtain detailed information about the extent of the destruction of health services hospitals in the temporarily occupied territories.

The worsening shortage and migration of mental health professionals further strain the system. The most noticeable decline has been observed in the number of doctors, social workers, and junior nurses compared to the period before the full-scale war. Moreover, this shortage has not been resolved in the past two years. This may be partly due to healthcare workers leaving the country to ensure greater safety and to continue their careers abroad, or seeking more secure and better-paid employment opportunities and a safe place for their families. With the ongoing security risks, the workload on the remaining mental health professionals continues to increase, especially in the western and central regions, where a large number of patients have been transferred. This poses a risk of burnout and further reduction in the workforce in the future [29, 30]. It is important to note that the ratio of medical personnel to psychosocial support staff continues to favor the biological treatment model. Consequently, the volume of psychosocial assistance provided during the hospital phase is likely limited, placing the primary burden of psychosocial care on other levels of the mental health support system.

The abovementioned and the ongoing nature of the war emphasises the necessity of restructuring the mental health care system to address not only immediate concerns but also long-term challenges. Strengthening crisis intervention, outpatient and community services with community mental health centres and mobile teams, complemented by a comprehensive set of interventions beyond the health sector while establishing a strong and efficient link with inpatient care integrated into general health care is essential to mitigate the strain on the mental healthcare system [13, 31].

Like any study, this research has its limitations. While self-reported data from hospital heads may introduce bias or inconsistencies, this limitation is minimized by the respondents’ expertise and the study’s emphasis on broader trends rather than individual cases. In addition, within the context of war, that is a focus of this study, this design also had considerable advantages (e.g., rapid data collection and analysis for monitoring purposes). Second, although only 41% of facilities participated in all three surveys, potentially limiting the representativeness of the findings, this remains the most comprehensive data available so far given the constraints of the war situation. Lastly, although a broader range of questions was intended, the war-related priorities of hospital heads restricted the scope of the study, limiting the depth of data collection.

## Conclusion

Our study highlights a significant rise in psychiatric hospitalizations two years into the full-scale war in Ukraine, surpassing both pre-war levels and those observed six months after the invasion. While hospitalizations for war trauma have increased, their proportion has remained stable, suggesting sustained demand for trauma-related care.

## Data Availability

No datasets were generated or analysed during the current study.
